# Anti-tumour activity and store operated calcium entry: new roles in immunology

**DOI:** 10.1002/emmm.201303129

**Published:** 2013-09-03

**Authors:** Kavisha Singh, Paul Rosenberg

**Affiliations:** Department of Medicine, Duke University Medical CenterDurham, NC, USA

**Keywords:** calcium, cytotoxic T cells, SOCE

See related article in EMBO Molecular Medicine http://dx.doi.org/emmm.201302989

Ca^2+^ signalling plays a critical role in almost every cell type of the immune system—T cells, B cells, NK cells, macrophages and mast cells. Intracellular Ca^2+^ signals in immunology have importance in both the short term and long term function of these cells. Short term functions like release of cytotoxic granules with inflammatory mediators, immune synapse formations and long term functions like cell differentiation, expression and proliferation are all regulated to various degrees by intracellular Ca^2+^ signals. Store operated calcium entry (SOCE) is one of the critical modulators of the Ca^2+^ signals that control these processes. The importance of SOCE in normal immune physiology has already been demonstrated in families with mutations in STIM1, STIM2 and Orai1 who demonstrate a severe immunodeficiency that causes bacterial, viral and fungal infections (Picard et al, 2009). Alterations in the immune system due to changes in Ca^2+^ homeostasis has spiked interest in studying the role of SOCE in another critical function of the immune system—anticancer immunity. Relatively little is known about SOCE and its role in oncology and the evidence gathered so far seems to suggest that it is both important in tumour detection as well as tumour growth (Prevarskaya et al, 2011). Mouse models have shown that STIM1 and Orai1 expression is critical for breast cell tumour migration and metastasis (Yang et al, 2009). Other work in human prostate cancer cells has shown that normal expression of Orai1 and SOCE are critical in maintaining the rate of apoptosis and thus, resistance to prostate cancer (Flourakis et al, 2010). Given the conflicting nature of the research in the role of SOCE in cancer immunity, it is necessary to appreciate that Ca^2+^ regulates a variety of complex immune functions, not all of which are cancer protective. More importantly, delineating the mechanisms of how SOCE affects intracellular functioning in various immune cell types is important for a more sophisticated understanding of how Ca^2+^ is important, since the research so far suggests that there is vast heterogeneity in the role of SOCE in subpopulations of cell types in the immune system.

» A few of the mechanisms in which cytotoxic T cells work against tumour cells is through the expression of FasL, release of cytolytic granules and secretion of cytokines such as IFNg and TNFa, which induce apoptosis of cancer cells. These processes are dependent on intracellular Ca^2+^ signals…«

Weidinger et al works on elucidating the role of SOCE in CD8^+^ T cells which are critical for their cytotoxic activity against tumour cells (Weidinger et al, 2013). A few of the mechanisms in which cytotoxic T cells work against tumour cells is through the expression of FasL, release of cytolytic granules and secretion of cytokines such as IFNg and TNFa, which induce apoptosis of cancer cells. These processes are dependent on intracellular Ca^2+^ signals and this paper provides evidence about how SOCE affects the functioning and physiology of many of these critical processes. The idea that intact SOCE is necessary for T-cell function, but not necessarily cell proliferation has been raised several times in previous research. Although the phenotype of SOCE mutants is similar to that seen in severe combined immunodeficiency (SCID), the immune cell populations in these patients seem to be largely preserved. Orai1^−/−^ T cells have been shown to proliferate normally despite reduction in function and many human families with immunodeficiency due to mutations in STIM1 show a normal T-cell count, despite the immunologic deficit in the normal functioning of their immune cells (Feske, 2009). This paper shows that the number of tumour specific cytotoxic cells in draining lymph nodes is identical in both wild type and STIM1/STIM2 deficient double knockout mice. In descriptions of human families with nonsense mutations in STIM1 that caused either reduced or absent SOCE, the T-cell repertoire was found to be normal or close to normal, with the more explicit deficit being in T-cell functioning (Picard et al, 2009). Similar evidence was found in a child with STIM1 deficiency that developed a fatal Kaposi's sarcoma who presented with a normal immunologic cell count (Byun et al, 2010). Although there is some variation in the counts of subpopulations of T cells in various human and mouse mutants with deficient SOCE, the overall evidence seems to suggest that the immunodeficiency caused by deficient SOCE is not a direct function of the number of T cells present.

The Weidinger paper raises several ideas about the mechanisms of cytotoxicity that are affected by mutations in STIM1 and STIM2 in cytotoxic T cells (Weidinger et al, 2013). One major mechanism that mediates normal cytotoxic function of T cells is the production of cytokines like IL-2, IFNg and TNFa that induce apoptosis in target cells. Previous work done on CD4^+^ T cells and T helper cells has shown that both STIM1 and STIM2 deficiency causes reduction in the production of these critical cytokines upon appropriate stimulation (Oh-Hora et al, 2008). However, a large proportion of the work has been limited to studying CD4^+^ cell populations. Given the role of cytotoxic T cells in antitumour immunity, proving the same in CD8^+^ cells is important. This paper provides evidence that SOCE is just as important in CD8^+^ T cells in mediating the normal production of these cytokines. The production of TNFa and IFNg are both reduced in STIM1/STIM2 double knockouts as compared to wild types, providing insight into the mechanism of the immunodeficiency of deficient SOCE in CD8^+^ T cells against cancer cells. The regulation of not just IFNg, but Type I interferons (IFNa & IFNb) by Ca^2+^ signalling and its effect on apoptosis has been demonstrated in human leukemic T-cell lines (Yue et al, 2012). Looking at the effect on Type I IFN production in CD8^+^ T cells with deficient SOCE may provide additional insight into the cytotoxicity of these cells, since IFNa has been implicated in maintaining the survival of memory CD8^+^ cells, which has direct implications for sustained antitumour immunity. A potential area for further research would be investigating the role of SOCE in Ca^2+^ release from secretory granules, especially given the Weidinger group's evidence about the release of Granzyme B and perforin from CD8^+^ cells and other published research suggesting that Orai1 channels on the membranes of secretory granules as well as secretory epithelia in breast cancer tissue have a critical role to play in calcium release (Cross et al, 2013; Dickson et al, 2012). The role of Orai1 in cell death in T-cell lines, by multiple mechanisms that could involve an increase in mitochondrial Ca^2+^ levels as well as regulation of NFAT signalling suggests that cell survival and apoptosis is another critical component to the story of antitumour immunity as regulated by store operated calcium entry (Srikanth and Gwack, 2013). Another mechanism represented is the expression of FasL on the surface of these tumour cells, which affects the interaction of the cytotoxic cells with their target tumour cells. The reduced expression of FasL on the surface of cytotoxic T cells post exposure to BTP-2 indicates that SOCE has a significant role to play in this mechanism of cytotoxicity. The expression of FasL is regulated by NFAT, whose nuclear translocation has been shown to be negatively affected in both STIM1 and STIM2 knockout helper T cells in mouse models. The sustained Ca^2+^ release required for the nuclear translocation of NFAT and its subsequent regulation of FasL expression lends support to the theory that intact SOCE as regulated by STIM1, STIM2, Orai1 can have transcriptional effects that affect the function of cytotoxic T cells against tumours.

» …SOCE is just as important in CD8^+^ T cells in mediating the normal production of these cytokines.«

Of note, this paper provides some theories about the individual importance of STIM1 and STIM2 in cytotoxic T-cell function against tumour cells. Their unpublished observations suggest that homozygous STIM1 deletions in CD8^+^ T cells have preserved cytotoxic activity, indicating that STIM2 is able to provide a degree of SOCE that is sufficient for effective antitumour immunity. Other research also suggests that STIM2 is a positive regulator of SOCE and not an inhibitor of STIM1, as was thought previously. STIM1 and STIM2 are present in different relative concentrations in the ER membrane, with STIM2 consistently present in smaller concentrations in most cell types. STIM1 and STIM2 also show differences in their calcium kinetics, with STIM2 being activated at basal ER Ca^2+^ concentrations and activating Ca^2+^ entry at a much earlier phase in store depletion in the ER. In T cells, it has been proven that Ca^2+^ influx is negatively affected by loss of STIM2, although to a much smaller degree than is seen in STIM1 loss of function models. However, STIM2 plays a role in compensating for the production of cytokines in these T cells when STIM1 is absent, suggesting that its importance should not be ignored. In addition, loss of STIM2 affected the ability of T cells to maintain NFAT concentrations in the nucleus, implicating that even STIM2 mutations could potentially impact transcriptional processes in cytotoxic T cells that are important for their activity against tumour cells (Oh-Hora et al, 2008). Given that this paper shows that STIM1/2 double knockout CD8^+^ T cells has reduced FasL expression, a downstream effect of NFAT, describing the roles of STIM1 and STIM2 independently is highly relevant to knowing how much they contribute to those intracellular processes. Since the majority of the evidence about the role of STIM2 comes from studies on CD4^+^ cells, additional work on CD8^+^ cell populations would be helpful before drawing inferences about the role of STIM1 and STIM2 in the double knockout model used in this study.

Overall, this paper provides important evidence for the role and mechanisms of SOCE mediated antitumour immunity in the CD8^+^ T-cell population, which is valuable information considering that a large volume of work has been restricted to other T-cell subtypes. It also draws an appropriate conclusion in terms of the clinical implications of the role of SOCE in antitumour immunity and using CRAC channel inhibitors as targets for anticancer therapy. An important role of SOCE in immunology that cannot be ignored is the role of Ca^2+^ in the proliferation and function of the suppressive Treg cell population, which is regulated by the NFAT-FoxP3 interaction (Oh-Hora et al, 2008). Given the body of research suggesting that SOCE is important in tumour genesis as well as activation and suppression of the immune system, the role of Ca^2+^ in the delicate balance of healthy immune functioning is complicated, to say the least. Further work is essential before drawing conclusions about the clinical effectiveness of drugs targeting SOCE, either as inhibitors or activators.
